# Perinatal (fetal and neonatal) astrocytoma: a review

**DOI:** 10.1007/s00381-016-3215-y

**Published:** 2016-08-27

**Authors:** Hart Isaacs

**Affiliations:** 1 Departments of Pathology, Rady Children’s Hospital, San Diego, CA USA; 2 University of California San Diego School of Medicine, La Jolla, CA USA

**Keywords:** Fetal astrocytoma, Neonatal astrocytoma, Perinatal astrocytoma, Intracranial hemorrhage, Congenital brain tumor

## Abstract

**Introduction:**

The purpose of this review is to document the various types of astrocytoma that occur in the fetus and neonate, their locations, initial findings, pathology, and outcome. Data are presented that show which patients are likely to survive or benefit from treatment compared with those who are unlikely to respond.

**Materials and methods:**

One hundred one fetal and neonatal tumors were collected from the literature for study.

**Results:**

Macrocephaly and an intracranial mass were the most common initial findings. Overall, hydrocephalus and intracranial hemorrhage were next. Glioblastoma (GBM) was the most common neoplasm followed in order by subependymal giant cell astrocytoma (SEGA), low-grade astrocytoma, anaplastic astrocytoma, and desmoplastic infantile astrocytoma (DIA). Tumors were detected most often toward the end of the third trimester of pregnancy.

**Conclusion:**

A number of patients were considered inoperable since their tumor occupied much of the intracranial cavity involving large areas of the brain. High-grade astrocytomas were more common than low-grade ones in this review. Fetuses and neonates with astrocytoma have a mixed prognosis ranging from as low as 20 % (GBM) to a high of 90 %. The overall survival was 47/101 or 46 %.

## Introduction

Glial cells are the supportive elements of the central nervous system (CNS) [22]. They include astrocytes, oligodendrocytes, and ependymal cells, and the corresponding tumors originating from these cells astrocytoma, oligodendroglioma, and ependymoma all of which are loosely called “glioma” [16, 22]. The term “glioma” is used interchangeably with astrocytoma to describe the more common subgroup of tumors [22].

Glioma (astrocytoma) is the leading CNS tumor in children. Low-grade gliomas are the leading pediatric CNS tumors overall and are responsible for approximately one third of cases [22, 63]. Higher grade gliomas occur less often and account for 7–11 % of cases of CNS malignancy beyond infancy [22, 28].

Low- and high-grade astrocytoma differs significantly in terms of their histological grading, site of origin, treatment, and prognosis. The various types of tumors within the group of low-grade astrocytoma are classified as WHO grade I and grade II [13, 39]. The major histological subtype in the perinatal period is the pilocytic astrocytoma, a WHO grade I tumor that is usually well circumscribed and has only a narrow margin of infiltration into the surrounding tissues [13]. Next in frequency is the fibrillary astrocytoma (WHO grade II), which has a tendency to be more infiltrative. High-grade astrocytomas are those graded III (e.g., anaplastic astrocytoma) or IV (e.g., glioblastoma) and comprise approximately 7–11 % of pediatric tumors [28]. Perinatal strocytomas vary from benign (low-grade) to malignant (high-grade) tumors [9, 18, 30, 32, 49, 51, 63, 69]. High-grade astrocytomas were more prevalent than low-grade ones in this review, 55 versus 46 % (Table 9).

Fetal and neonatal brain tumors are rare comprising only 0.5–1.5 % of all childhood CNS tumors [68]. Astrocytoma is not only the main neuroglial tumor but also second in incidence following intracranial teratoma [12, 24, 30–32, 35, 36, 55, 76, 82, 90]. Perinatal astrocytomas occur most often outside the posterior cranial fossa and above the tentorium. Cerebral hemisphere is the main location where they tend to be large, involve more than one lobe, and displace the lateral and/or third ventricles [24, 30, 35, 36, 55, 56, 81, 82]. Intracranial mass, macrocephaly, hydrocephalus, and hemorrhage are the most common initial imaging findings [23, 27, 30, 32, 38, 51] (Tables 2, 3, 4, 5, 6, 7, and 8). However, the leading presentation is an intracranial mass detected on routine prenatal sonograms [23, 27, 30, 32, 81]. Differential diagnosis of a brain tumor includes vascular malformation, infarction, or hemorrhage [21, 47, 83, 84].

## Materials and methods

Retrospective review of 101 cases including 57 fetuses and 44 neonates with astrocytoma was performed. They were collected from the literature, mostly from the archives of the National Library of Medicine (PubMed MEDLINE) and from our institution. The study was confined to fetuses with astrocytoma that were detected prenatally by imaging studies or discovered at birth and neonates with tumors occurring during the first 2 months of life. Only patients with adequate clinical and pathological data and where outcomes of pregnancy and survival were given were accepted for review. The following data were collected and tabulated: maternal age, gravida, initial presentation, and obstetric management, and the fetal data—sex of the fetus, weeks of gestation, birth weight, tumor location and dimensions, histology and grade, treatment, and outcome. Diagnosis of the various tumors was established in patients included in the study by histological confirmation of biopsy or surgical and/or postmortem specimens. Time period of patient accrual was from 1970 to 2015. Length of follow-up varied from 1 week to 1 year.

## Results

One hundred one fetuses and neonates, presented with astrocytoma (Tables 1, 2, 3, 4, 5, 6, 7, 8, and 9). Most tumors were detected during the third trimester of pregnancy. They varied from low-grade, I and II, to high-grade malignancies (grades III and IV) (Tables 2, 3, 4, 5, 6, 7, and 8). High-grade astrocytomas were more prevalent than lower grade ones, a ratio of 1.5:1. Glioblastoma was the major neoplasm, 45 or 44.6 %. Next were subependymal giant cell astrocytoma 18 (17.9 %), followed by ten examples of each anaplastic astrocytoma (9.9 %), low-grade (I–II) astrocytoma (9.9 %), and desmoplastic infantile astrocytoma/ganglioglioma (9.9 %); the two less common tumors were astroblastoma, 5 (4.9 %), and gemistocytic astrocytoma, 3 (2.97 %) examples.

**Table 1:** Distribution of 101 perinatal astrocytomas

Tumor	Number (%)
Glioblastoma	45 (44.6)
Subependymal giant cell astrocytoma	18 (17.9)
Low-grade, I–II, astrocytoma	10 (9.90)
Anaplastic astrocytoma	10 (9.90)
Desmoplastic infantile astrocytoma/ganglioglioma	10 (9.90)
Astroblastoma	5 (4.90)
Gemistocytic astrocytoma	3 (2.97)
Total	101 (100)

**Table 2:** Fetal and neonatal glioblastoma (*n* = 45)

Female/male	21/17 = 1.23^a^
Fetuses^b^	28
Neonates^c^	17
Gestation (weeks)	36, range 29–40
Birth weight (g)	3152, range 1400–4270
Maternal history	
Maternal age (years)	27 years, range 17–38
Gravida	G_1_ 9, G_2_ 2, G_4_ 1, G_5_ 2
Delivery	
Vaginal	26
Cesarian section	19
Termination of pregnancy	3
Initial findings	
Macrocephalus	28
Hydrocephalus	27
Intracranial mass on imaging	26
Intracranial hemorrhage	13
Bulging anterior fontanel	13
Stillbirth	5
Seizures	3
Dystocia	3
Increasing irritability	3
Hydramnios	3
Vomiting	3
Eye signs^d^	3
Facial palsy	3
Hemiparesis	3
Hypotonia	2
Breech presentation	2
Location	
Cerebral hemisphere, NOS^e^	34
Thalamus	3
Cerebellum and cerebellar-pontine angle	2
Cerebellum and basal ganglia	2
Midline suprasellar	1
Hypothalamus	1
Midline replacing most of brain	1
Cerebral hemisphere replaced by tumor	1
Tumor filled intracranial cavity replacing brain	1
Septum pellucidum and adjacent ventricle	1
Tumor characteristics	
Brain weight (g)	597, range 250–850
Tumor weight (g)	20, 5.8^f^
Tumor greatest dimension (cm)	9.6, range 5–11
Treatment	
Patients treated	16 (35.6)
Patients not treated	29 (64.4)
Survival with surgical resection alone	2/6
Survival with SR + CT^c^	4/4
Survival with SR + XRT	2/2
Survival with SR + CT + embolization	1/1
Survival with SR + CT + XRT	0/2
Survival with CT	0/1
Outcome	
Fetal survival	2/31 (6.0)
Neonatal survival	7/14 (50)
Patients treated survived	9/16 (56)
Patients not treated survived	0/29
Overall survival	9/45 = 20 %

Cases selected from the literature

*CT* chemotherapy, *NOS* not otherwise specified, *SR* surgical resection, *XRT* radiation therapy

^a^Sex not stated in seven patients

^b^Tumor discovered prenatally or on the first day of life

^c^Tumor discovered within the first 2 months of life

^d^Eye signs: setting sun, doll’s eyes, nystagmus

^e^Cerebral hemisphere locations: parietal 3, frontal 2, and one each: parietal-occipital, frontal-parietal

^f^Tumor weights were given only in two patients

^g^Two of nine survivors were alive with disease

**Table 3:** Fetal and neonatal subependymal giant cell astrocytoma (*n* = 18)

Male/female	14/4 = 3.5
Fetuses^a^	11
Neonates^b^	7
Gestation (weeks)	35, range 22–40
Birth weight (g)	2653, range 2100–3600
Family history of tuberous sclerosis	1
None	9
Family history not stated	8
Maternal history	
Maternal age	27 years, range 17–38
Gravida	G_1_ 6, range G_1_–G_6_
Delivery	
Vaginal	15
Cesarian section	2
Termination of pregnancy	1
Initial findings	
Cerebral hemisphere mass	14
Hydrocephalus	11
Concomitant cardiac rhabdomyoma	10
Seizures	3
Macrocephaly	2
Other^c^	10
Location	
Subependyma lateral ventricle adjacent to foramen of Monro	10
Cerebral hemisphere, NOS	4
Subependyma lateral ventricle, basal ganglia, and/or thalamus	2
Within a lateral ventricle	2
Tumor characteristics	
Tumor greatest dimension (cm)	4, range, 2–9
Treatment	
Patients treated	12^d^
Patients not treated	5^e^
Survival with surgery alone	9/11 (81.8)^f^
Survival SR + CT	2/2 (100)^g^
Patients lost to follow-up	1
Outcome	
Fetal survival	6/11 (54.5)
Neonatal survival	5/6 (83.3)^h^
Patients treated survived	11/12 (91.7)
Patients not treated survived	1/6 (16.7)
Overall survival^h^	12/17 = 70.6 %

Cases selected from the literature

*CT* chemotherapy, *NOS* not otherwise specified, *SR* surgical resection, *XRT* radiation therapy

^a^Tumor discovered prenatally or on the first day of life

^b^Tumor discovered within first the 2 months of life

^c^Other initial findings, one each: fetal hydrops, dystocia, hydramnios, lethargy, hypotonia, opisthotonus, abnormal eye movements, vomiting, hemiparesis, cyanosis, respiratory distress

^d^Patients treated: seven fetuses, five treated

^e^Patients not treated: four fetuses, five neonates

^f^( ) = percent

^g^SR + CT; one fetus and one neonate

^h^One neonate was lost to follow-up

**Table 4:** Fetal and neonatal “low-grade” astrocytoma (grades I and II) (*n* = 10)

Male/female	6/4 = 1.5
Fetuses^a^	3
Neonates^b^	7
Gestation (weeks)	39, range 37–40
Birth weight (g)	2902, range 2000–3990
Maternal history	
Maternal age (years)^c^	
Gravida^c^	
Delivery	
Vaginal	9
Cesarian section	1
Initial findings^d^	
Mass discovered on imaging	8
Intracranial hemorrhage	3
Bulging anterior fontanel	3
Macrocephalus	2
Decreased movement of extremity	2
Quadraparesis	2
Hypotonia	2
Breech presentation	2
Eye signs	2^e^
Hemiparesis	2
Breech presentation	2
Other findings	7
Location	
Spinal cord	5
Cerebral hemisphere	3
Optic nerve	2
Tumor characteristics^f^	
Treatment	
Patients treated	10
Patients not treated	0
Survival with surgical resection alone	9/9
Survival with SR + CT^c^	1/1
Outcome	
Fetal survival	2/3
Neonatal survival	7/7
Patients with spinal cord tumors who survived with significant disabilities	3/4 (75)
Overall survival	9/10 = 90 %

Cases selected from the literature

*CT* chemotherapy, *SR* surgical resection

^a^Tumor discovered prenatally or on the first day of life

^b^Tumor discovered within the first 2 months of life

^c^Maternal age and gravida were given only in one of ten reports

^d^Other initial findings, one each: hydrocephalus, dystocia, head tilt, seizures, irritability, poor feeding, vomiting

^e^Optic nerve tumor eye associated signs: microphthalmia, orbital cyst, proptosis, exophthalmia

^f^Tumor characteristics such as brain weight, tumor weight, and tumor dimensions were not listed in the ten reports reviewed

**Table 5:** Fetal and neonatal anaplastic astrocytoma (*n* = 10)

Female/male	5/4 = 1.25^a^
Fetuses^b^	7
Neonates^c^	3
Gestation (weeks)	37, range 31–40
Birth weight (g)	3873, range 3100–4700
Maternal history	
Maternal age (years)	28 years, range 20–33
Gravida	G_1_ 1, G_2_ 1, NS 8
Delivery	
Vaginal	7
Cesarian section	3
Termination of pregnancy	1
Initial findings	
Intracranial mass on imaging	7
Macrocephalus	6
Hydrocephalus	6
Intracranial hemorrhage	3
Bulging anterior fontanel	3
Seizures	2
Irritability	2
Other findings^d^	5
Location	
Cerebral hemisphere^e^	10/10 (100)
Tumor characteristics	
Tumor greatest dimension (cm)	9 cm, range 6–12
Weight^f^	
Treatment	
Patients treated	6/10 (60)
Patients not treated	4/10 (40)
Survival with surgical resection alone	2/2 (100)
Survival with SR + CT^f^	3/3 (100)
Survival with SR + CT + XRT	1/1 (100)
Outcome	
Fetal survival	5/7 (71.4)
Neonatal survival	1/3 (33.3)
Patients treated survived	6/6 (100)
Patients not treated survived	1/1 (100)
Overall survival	6/10 = 60 %^g^

Cases selected from the literature

*CT* chemotherapy, *SR* surgical resection, *XRT* radiation therapy

^a^Sex not stated in one patient

^b^Tumor discovered prenatally or on the first day of life

^c^Tumor discovered on the first 2 months of life

^d^Other presenting findings, one each: dystocia, vomiting, palsy, hemiparesis, no recognizable cerebral structures

^e^Cerebral hemisphere locations: one each, parietal, parietal-occipital, temporal, both cerebral hemispheres; cerebral hemisphere site, not specified, 6

^f^Tumor weights were given only in four patients

^g^Three survivors were alive with disease

**Table 6:** Fetal and neonatal desmoplastic infantile astrocytoma/ganglioglioma (*n* = 10)

Female/male	5/4 = 1.25^a^
Fetuses^b^	1
Neonates^c^	9
Gestation (weeks)	37, range 34–40
Maternal history^d^	
Delivery	
Vaginal	8
Cesarian section	2
Initial findings	
Macrocephaly	9
Intracranial mass on imaging	7
Bulging anterior fontanel	2
Seizures	2
Increasing irritability	2
Other findings	10^d^
Location	
Cerebral hemisphere	10
Frontal-parietal-temporal	5
Parietal-temporal	1
Parietal-occipital	1
Frontal-temporal	1
Bilateral cerebral hemispheres	1
Cerebral hemisphere replaced by tumor	1
Tumor characteristics	
Tumor greatest dimension (cm)	11.8, range 11–13
Tumor diagnoses	
Desmoplastic infantile ganglioglioma^e^	7
Desmoplastic infantile astrocytoma^f^	3
Treatment	
Patients treated	10
Survival with surgical resection alone	8/9 (89)^h^
Survival with SR + CT^g^	0/1
Outcome	
Fetal survival	1/1
Neonatal survival	7/9 (77.8)
Patients treated survived	8/10 (80)
Overall survival	8/10 = 80 %

Cases selected from the literature

*CT* chemotherapy, *DIG* desmoplastic infantile astrocytoma, *GBM* glioblastoma, *SR* surgical resection

^a^In one patient sex was not stated

^b^Tumor discovered prenatally or on the first day of life

^c^Tumor discovered within the first 2 months of life

^d^Gravida and maternal age were not mentioned in the ten reports reviewed

^e^Other initial findings, one example each: hydrocephalus, vomiting, setting sun eye sign, lethargy, hemiparesis, hypotonia, malaise, intracranial hemorrhage, breech presentation, dystocia

^f^Desmoplastic infantile ganglioglioma: ganglion cells present in addition to astrocytic cells

^g^Desmoplastic infantile astrocytoma: astrocytic cells present; ganglion cells not present

^h^One patient was operated upon at age 2 months for removal of DIG. Subsequently 8 years later, the tumor recurred as GBM [2]

**Table 7:** Fetal and neonatal astroblastoma (*n* = 5)

Male/female	4/1 = 4
Fetuses^a^	4
Neonates^b^	1
Gestation (weeks)	35, range 31–39
Birth weight (g)	2725, range 2500–2950
Maternal history	
Maternal age^c^	–
Gravida^c^	–
Delivery	
Vaginal	3
Cesarian section	2
Initial findings	
Mass discovered on imaging	5
Macrocephaly	4
Hemorrhage	3
Hydrocephalus	2
Bulging anterior fontanel	1
Respiratory distress	1
Location	
Cerebral hemisphere^d^	5
Cerebral hemisphere replaced by tumor	2
Tumor characteristics	
Tumor greatest dimension (cm)	10, range 9–11
Treatment	
Patients treated	4
Patients not treated	1
Survival with surgical resection alone	0/2
Survival with SR + CT	2/2 (100)
Outcome	
Fetal survival	1/3 (33.3)^e^
Neonatal survival	1/1 (100)
Patients treated survived	2/4 (50)
Patients not treated survived	0/1
Overall survival	2/5 = 40 %

Cases selected from the literature

^a^Tumor discovered prenatally or on the first day of life

^b^Tumor discovered within the first 2 months of life

^c^Maternal age and gravida were given for patient no. 5 (age 29, G_4_, P_3_)

^d^Cerebral hemisphere locations: cerebral hemisphere ( frontal lobe 2, cerebral hemisphere, site NOS, 3)

^e^Fetus lost to follow-up (presumed dead)

**Table 8:** Fetal and neonatal gemistocytic astrocytoma (*n* = 3)

Female/male	2/1
Fetuses^a^	3
Neonates^b^	0
Gestation (weeks)	35, range 32–40
Birth weight (g)	3523^c^
Maternal history	
Maternal age (years)	29, range 19–36
Gravida	G_1_, G_3_
Delivery	
Vaginal	0
Cesarian section	3
Initial findings	
Macrocephalus	3
Hydrocephalus	3
Intracranial mass discovered on imaging	2
Intracranial hemorrhage	2
Bulging anterior fontanel	1
Seizures	1
Dystocia	1
Irritability	1
Vomiting	1
Location	
Cerebral hemisphere, occipital lobe	1
Cerebral hemispheres replaced by tumor	1
Tumor filled one half of intracranial cavity	1
Tumor characteristics	
Brain weight (g)	654^c^
Tumor weight (g)	
Tumor greatest dimension (cm)	
Treatment and survival	
Patients treated	1 (1/3)
Patients not treated	2 (0/3)
Survival with surgical resection + chemotherapy	1 (1/1)^d^
Outcome	
Fetal	1/3
Patients treated survived	1/1
Patients not treated survived	0/2
Overall survival	1/3 = 33 %

Cases selected from the literature

^a^Tumor discovered before birth or on the first day of life

^b^Tumor discovered during the first 2 months of life

^c^Given for one patient only

^d^Combination chemotherapy using cisplatinum and vincristine. Patient had a recurrence at age 10 months

**Table 9:** Fetal and neonatal astrocytoma: histological type, grade versus survival (*n* = 101)

Tumor	Grade	Number	Survival (%)
Glioblastoma	IV	45 (44.6)	9/45 (20)
Subependymal giant cell astrocytoma	I	18 (17.9)	11/18 (61)
Low-grade, I–II, astrocytoma	I–II	10 (9.90)	9/10 (90)
Anaplastic astrocytoma	III	10 (9.90)	6/10 (60)
Desmoplastic infantile astrocytoma/ganglioglioma	I	10 (9.90)	8/10 (80)
Astroblastoma	?^a^	5 (4.90)	3/5 (60)
Gemistocytic astrocytoma	II	3 (2.97)	1/3 (33.3)
Total		101 (100)	47/101 (46.5)

^a^Grade has not been assigned for this tumor (39,54)

Overall, the main presenting findings were macrocephaly and an intracranial mass followed by hydrocephalus and intracranial hemorrhage (Tables 1, 2, 3, 4, 5, 6, 7, 8, and 9). Nine percent (5/57) of the affected fetuses were stillborn. The overall survival for both fetuses and neonates was 47/101 or 46.5 %.

### Glioblastoma malignant astrocytoma grade 4 (GBM)

Arises from the cerebral hemispheres and basal nuclei of fetuses, stillbirths, and infants [21, 23, 24, 30, 38, 43, 49, 68, 72]. It is the leading form of astrocytoma in this and in other similar perinatal studies [12, 15, 30, 31, 68]. GBM comprised almost half of the cases here (Tables 1, 2, and 9). An echogenic mass occupying much of one or both hemispheres accompanied by a shift of midline structures, obstructive hydrocephalus, and hemorrhage were the main prenatal ultrasound findings [21, 23, 38, 89, 93] (Table 2). Serial imaging studies showed that tumor growth developed rapidly over a surprisingly short period of time. Intracranial hemorrhage contributed also to the tumor’s rapid size increase [38, 89]. Dystocia occurred when there was a large space occupying intracranial mass. Cephalocentesis (cranial perforation) may be required for removal of the tumor before vaginal delivery could occur [1]. Large head circumference, hydrocephalus, and a bulging anterior fontanel were the main presenting findings in the neonate.

#### Pathology

**Fig. 1:**
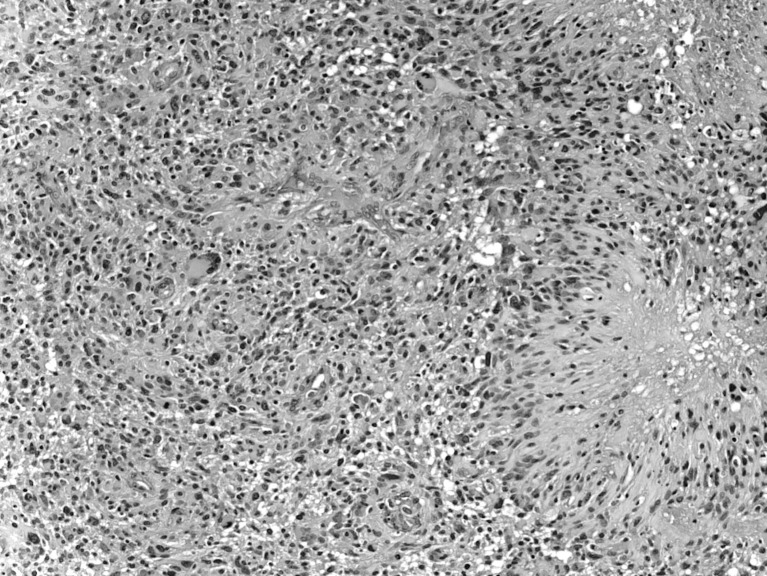
GBMs showing hypercellularity, marked pleomorphism, tumor giant cells, microvascular proliferation, and “palisading necrosis” (neoplastic spindle-shaped cells form palisades around central necrotic foci)

The cerebral hemisphere was the most common site of origin, namely, 34/45 or 76 % (Table 2). On gross inspection, many GBMs were large with soft pale-gray, hemorrhagic, and necrotic areas [35, 42, 82, 84]. Microscopically, they showed hypercellularity, marked pleomorphism, tumor giant cells, microvascular proliferation, and “palisading necrosis” (neoplastic spindle-shaped cells form palisades around central necrotic foci) [11, 13, 30, 39] (Fig. 1). GFAP and vimentin are immunoreactive. Some other findings include a strong nuclear expression of p53 and 10q deletions. High-grade astrocytomas were more prevalent than low-grade ones in this review.

Fetal and neonatal GBMs are genetically different from their adult counterparts and show a low frequency of known genetic defects [11].

#### Treatment and outcome

Fetuses with GBM may be born moribund or die soon after birth [1, 21, 27, 30, 49]. The prognosis for young patients with this tumor is discouraging low (Table 2). The overall survival rate was only 9 of 45 or 20 % (Tables 2 and 9). Nevertheless, cure was not always out of the question. All six patients who had surgical resection and chemotherapy [10, 11, 46, 50], one with surgical resection, embolization, and chemotherapy [74], and two of six who received surgery as the only treatment, survived [93] (Table 2). Neonatal survival of 50 % was higher than fetal, which was only 6.5 %.

*Subependymal* *giant cell astrocytoma* (SEGA) is one of the unique forms of astrocytoma. It occurs in brains of patients with *tuberous sclerosis* (TSC) and in those without the disease. Most are found during the first and second decades of life and only occasionally in the perinatal period [30, 31, 51, 56, 58–60, 63] (Table 3).

TSC is an autosomal-dominant inherited condition characterized by highly variable clinical manifestations including seizures, mental retardation, skin lesions, and hamartomas affecting multiple organ systems such as the heart, brain, eye, and kidney [33, 60, 73, 89] (Table 3). Brain lesions consist of subependymal nodules, white matter heterotopias, cortical tubers, and SEGA [73]. Cortical tubers and subependymal nodules are much more common than SEGA [31]. Tubers are situated throughout the white matter whereas subependymal nodules characteristically are found in or near the ventricles. The former are composed of firm, focal areas consisting of large eosinophilic cells with prominent nuclei suggesting features of both neurons and astrocytes [13, 33, 73]. Subependymal nodules have a histological appearance similar to SEGA. In some instances, there is really no well-defined cytologic difference between the two. Moreover, serial imaging and biopsy studies reveal transitions of subependymal nodules to SEGA [73]. Apparently, size of the lesion does not seem to be helpful in distinguishing between the two.

Fetuses and neonates with TSC present more often clinically with their cardiac problems, namely uncontrollable arrhythmias and intracavitary outflow obstruction due to rhabdomyoma(s), rather than manifestations of their CNS pathology [30, 31, 33, 89]. For example, 11 of 18 fetuses and neonates in the study here had 1 or more cardiac rhabdomyoma. Newborns diagnosed initially with SEGA may go on to develop other manifestations of TSC [33].

Table 3 shows that males are affected more often than females, a ratio of 3.5 to 1. Of the 18 patients with SEGA a family history of TSC was given only in one; however, a history of this disease was not mentioned in eight patients. Hydrocephalus and an intracranial mass were the main presenting findings (Table 3). SEGAs appeared on sonograms as subependymal echogenic nodules situated in the walls of lateral ventricles typically near the foramen of Monro. The tumor was nodular and of mixed signal or iso-dense to the brain, enhancing densely with contrast on CT scan. Calcifications are noted often within the tumor [51, 56, 59, 63].

#### Pathology

**Fig. 2:**
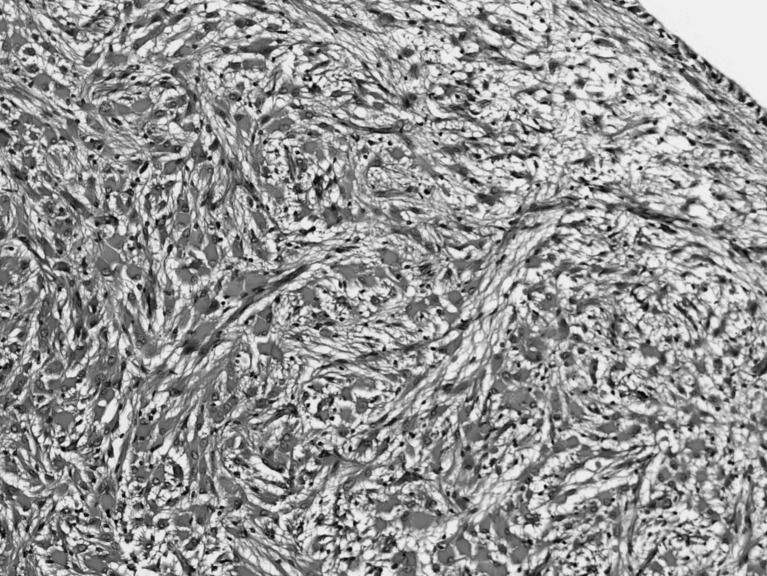
Microscopic examination revealing large astrocytic cells with abundant pink staining cytoplasm, slightly vesicular nuclei, and prominent nucleoli

SEGAs occurred as outgrowths from within or beneath the walls of lateral ventricles. They obstructed the foramen of Monro or blocked the third ventricle producing hydrocephalus [30, 51, 56, 58]. Cerebral hemisphere was the most common site of origin occurring all 18 patients here (Table 3). Ten were located near or within the foramen of Monro obstructing it. Microscopic examination reveals large astrocytic cells with abundant pink staining cytoplasm, slightly vesicular nuclei, and prominent nucleoli (Fig. 2). Cells are surrounded by numerous fibrillary processes. The tumors are variably immunoreactive with GFAP, S-100, and β-tubulin, suggesting perhaps a mixed glial-neuronal phenotype [39]. Although histologically SEGA resembles gemistocytic astrocytoma, the latter is more infiltrative and located within the parenchyma rather than a well-demarcated, exophytic intraventricular growth. Moreover, SEGA cells tended to be larger than neoplastic gemistocytes, which also usually lack calcifications [13, 73].

#### Treatment and outcome

Twelve of 18 SEGA patients were treated; 5 were not. One nontreated neonate was lost to follow-up. Nine of 11 infants treated with either surgical resection alone or surgery plus evorolimus chemotherapy survived [40, 51, 56, 60, 64]. Overall survival rate was 70.6 % (Table 3). Fetal deaths were five times greater than neonatal. Treatment of SEGA is discussed in detail elsewhere [92].

*Low* *-grade astrocytoma*—*pilocytic astrocytoma*, *grade I*, arises throughout the neural axis. The spinal cord, cerebral hemisphere, and optic nerve were the tumor locations in this review. Half occurred in the spinal cord [17, 53, 77], three in the cerebral hemisphere [7, 44, 70, 83, 90], and two in the optic nerve [7, 44] (Table 4). Intracranial mass on prenatal imaging was the most common presentation in the fetus whereas a spinal mass and paralysis were the main ones in the neonate (Table 4).

#### Pathology

**Fig. 3:**
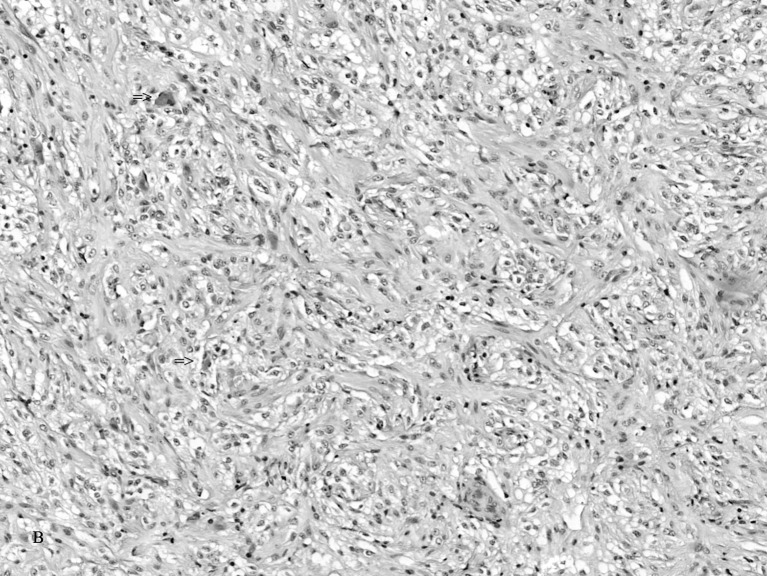
The tumor consisting of small bipolar and stellate-shaped cells with scanty processes forming loose and compact areas and microcysts

Low-grade 1 *pilocytic astrocytoma* exists on the benign end of the histologic spectrum. The tumor consists of small bipolar and stellate-shaped cells with scanty processes forming loose and compact areas and microcysts [13, 39] (Fig. 3). Rosenthal fibers and eosinophilic granular (cytoid) bodies are characteristically but variably present. Anaplastic changes consisting of cellular and nuclear pleomorphism, nuclear hyperchromatism, neoplastic giant cells, and bizarre mitoses are absent. Mitoses are uncommon. Astrocytoma cells react strongly with GFAP and negatively with synaptophysin, neurofilament, desmin, cytokeratin, and epithelial membrane antibodies [9, 13, 30, 39].

Optic pilocytic astrocytoma originates as a fusiform or globular mass arising from the optic nerve [7, 9]. The tumor produces a dumbbell-shaped mass with cyst formations in both intraorbital and intracranial segments of the nerve. There is a significant association between optic pathway pilocytic astrocytoma and *neurofibromatosis type 1* approaching 50 % in some series [9, 30, 34, 67]. This association foretells a poor prognosis.

#### Treatment and outcome

All three patients with low-grade I and II cerebral astrocytoma treated by surgical resection as the only form of therapy were cured [70, 83, 90]. Both neonates with optic astrocytoma survived, one received surgery only and the other surgery plus vincristine and carboplatin [7, 44]. There were five patients with spinal cord tumors, and of these, four survived. Death occurred in a 3120-g, 37-week gestation male fetus who was the product of a breech presentation. He received a subtotal resection for a spinal cord, T8–T11, tumor. Subsequently, the baby developed paralysis of both upper and lower extremities and died at age 8 weeks [17]. Overall survival for the entire low-grade astrocytoma group was 9/10 or 90 %, which is the highest figure recorded for the entire review (Tables 1, 2, 3, 4, 5, 6, 7, 8, and 9). Unfortunately, three of the four patients with spinal cord tumors who survived had serious residual disabilities. On the other hand, two with low-grade cerebral astrocytoma were left without significant ones. More detailed treatment of low-grade astrocytoma is discussed elsewhere [16, 26, 65, 79].

*Anaplastic* *astrocytoma* occurs less often in older children than the low-grade-astrocytoma whereas in the fetus and neonate, the incidence of the two is about the same (Tables 4 and 5). The term “anaplastic astrocytoma” refers to those tumors of intermediate-grade malignancy corresponding to the WHO classification grade III [13, 39].

#### Pathology

**Fig. 4:**
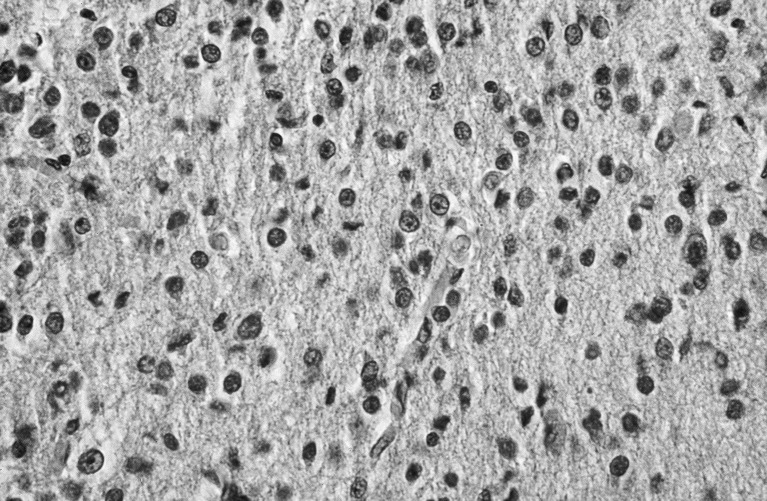
Anaplastic astrocytomas showing cytoplasmic and nuclear pleomorphism, hypercellularity, mitotic activity to a degree, but lack the pallisading necrosis or vascular proliferation of GBM

All ten anaplastic astrocytomas were located in the cerebral hemispheres [14, 18, 19, 27, 57, 69, 80, 94] (Table 5). Microscopically, they show cytoplasmic and nuclear pleomorphism, hypercellularity, mitotic activity to a degree, but lack the pallisading necrosis or vascular proliferation of GBM [18, 19, 27, 69, 78, 80, 94] (Fig. 4). However, one exception was a specimen from a stillborn fetus (therapeutic termination) whose tumor was discovered by ultrasound at 31-week gestation; intracranial hemorrhage and vascular proliferation were found along with pleomorphism, hypercellularity, and mitotic activity, suggesting perhaps an early in utero transformation to GBM [27]. GFAP and vimentin antibodies are reactive, but NSE, synaptophysin, neurofilament, desmin, cytokeratin, and epithelial membrane antigen are not.

#### Treatment and outcome

Six of ten patients with cerebral anaplastic astrocytomas were treated by several modalities: (1) surgical resection as the only form of therapy [18, 69], (2) surgery plus chemotherapy [19, 80, 94], and (3) surgery followed by chemotherapy and irradiation [78]. Five of six survived no matter what form of therapy was given (Table 5). Of the four patients who were not treated, one survived. Overall survival was 6/10 or 60 %.

*Desmoplastic* *cerebral astrocytoma/desmoplastic cerebral ganglioglioma of infancy* (*DIAGA*) occurs most often during the first year of life than at any other time [2, 4, 20, 25, 30, 45, 47, 62, 71, 81, 83, 86]. The diagnosis was made in this review in nine neonates and in one fetus (Table 6). There was a 9:1 male predominance. Tumors in seven patients were detected prenatally by ultrasound and appeared typically as a large cystic mass with a solid component situated within a cerebral hemisphere; most extended peripherally into the dura [20, 30, 45, 75, 86]. A large, intracranial hemorrhage was present also in the fetal patient obscuring the tumor and making diagnosis difficult [83].

An ongoing discussion in the literature exists regarding nosology and grading of this group of tumors [30, 39]. The question is raised whether infantile desmoplastic cerebral ganglioglioma (DIAG) and desmoplastic cerebral astrocytoma (DIA) represent the same histologic entity or two different conditions. During the perinatal period, their clinical features, pathology, treatment, and outcome are almost the same.

In addition, there is some disagreement about the WHO grading classification [30, 39]. The initial benign histology and clinical findings in the ten patients in this review are more consistent with a grade I–II tumor rather than a high-grade one. Nevertheless, it should be mentioned that some DIAGAs reported in some older children and adults have been diagnosed malignant [2, 62].

#### Pathology

**Fig. 5:**
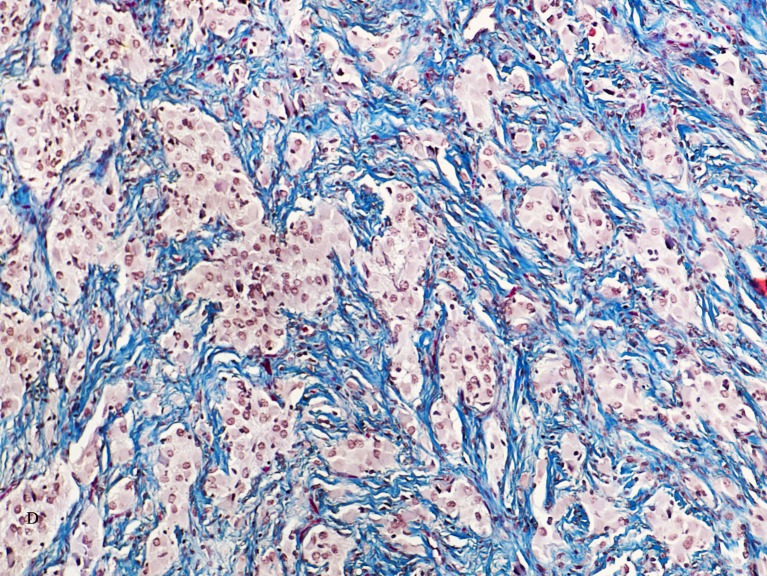
Characteristic histologic findings showing a cellular, spindle cell tumor with a prominent collagenous (desmoplastic, fibrous) stroma composed of bipolar and spindle-shaped cells having an eosinophilic cytoplasm and cells with long processes. The prominent, blue collagen component is shown by the Mallory trichrome stain.

Characteristic histologic findings are a cellular, spindle cell tumor with a prominent collagenous (“desmoplastic,” fibrous) stroma composed of bipolar and spindle-shaped cells having an eosinophilic cytoplasm and cells with long processes [20, 30, 45, 83, 86] (Fig. 5). Astrocytic cells form bundles and nests separated by thick bands of collagen [4, 30, 45, 83, 86]. Lymphocytes are variably present. Reticulin stain shows thick fibers surrounding nests of individual cells. Tumor cells and their processes react with GFAP and collagen fibers stain strongly blue with Masson’s trichrome [20, 30]. Ganglion cells were present in the six DIAG tumors but not identified in the four DIAs (Table 6).

#### Treatment and outcome

Eight of ten patients who were treated by surgery alone were cured [2, 4, 25, 47, 81, 88]. The male fetus listed in Table 6 was operated upon at age 2 months for removal of a DIAG. Eight years later, he died; the tumor recurred as GBM [62].

*DIAGA* is one of the astrocytoma groups usually associated with a relatively good prognosis overall provided that a complete resection was done; there was an 80 % survival for this group of tumors [4, 20, 45, 86] (Table 6).

*Astroblastoma* is a rare, controversial, supratentorial, tumor first described by Bailey and Bucy in 1930 [3]. The existence of this neoplasm has been debated ever since. The exact cell of origin has not been established [61]. Currently, it is placed in the WHO classification as a neuroepithelial tumor of uncertain origin [39]. It is exceedingly rare for less than five prenatal cases have been reported [5, 29, 35, 61, 66, 87] (Table 7).

#### Pathology

The dominant histologic feature is the characteristic perivascular pseudorosette [8, 66]. Two definite histological types of astroblastoma are described: low-grade and high-grade [8]. The low-grade lesion is a better differentiated tumor with more benign histologic findings, and the high-grade type has more anaplastic features and a poorer prognosis [8]. Low-grade astroblastoma consists of orderly perivascular pseudorosettes, having a papillary appearance, minimal cellular atypia, rare mitoses, and no microvascular proliferation. High-grade lesions show loss of the typical papillary architecture, areas of solid growth, increased cellularity with nuclear atypia, increased mitotic activity, and microvascular proliferation [8, 52, 85]. As one would anticipate low-grade, differentiating astroblastoma has a better prognosis than the anaplastic type [52]. At present, a WHO grade has not been assigned to this tumor [52].

The main entity in the differential diagnosis of astroblastoma is the well-differentiated ependymoma [13, 61, 66]. Clear spaces are present between the astroblastoma pseudorosettes whereas the ependymoma has a more compact architecture. There are differences in nuclear characteristics and thickness of the perivascular cytoplasmic processes as well. Astroblastomas exhibit broad foot plates opposed to tapering processes as seen in ependymomas [61]. Some tumors may resemble papillary meningioma or even a choroid plexus tumor. The main difference here is that astroblastoma is immunoreactive with GFAP whereas the meningioma is not; choroid plexus papilloma is focally GFAP positive [13].

#### Treatment and outcome

Treatment of astroblastoma is unsettled, largely because the rarity of the tumor precludes definitive therapeutic studies. Anaplastic histology is an important prognostic factor. Surgical resection is regarded as the preferred treatment modality; it plays a major role in managing the well-differentiated astroblastoma [52]. The precise role for irradiation and chemotherapy has not been well defined since only a few cases have been published to date [85]. However, these procedures could be considered in the treatment of the anaplastic type [52]. Prognosis is further complicated by the potential of the astroblastoma converting a more malignant form of glioma, namely GBM [8]. The clinical course is unpredictable.

*Gemistocytic* *astrocytoma* (*GA*) is a member of the diffuse astrocytoma group [13, 39, 41, 54]. It is much more common in older individuals where survival rates are higher than those in the fetus and neonate. Literature review uncovered only three examples of fetal GA all detected by prenatal ultrasound [6, 48, 54] (Table 8).

#### Pathology

Certain histologic criteria are required to make the diagnosis of GA. The main one is that a significant number of neoplastic gemistocytic astrocytes must be present in the tumor, at least 20 %, since collections of well-differentiated GA cells can mimic reactive astrocytes [39]. Although the tumor resembles SEGA, the GA cells are smaller and have a more prominent, eosinophilic cytoplasm containing an eccentric nucleus and a small nucleolus. Cells are surrounded by thick and thin cell processes. Tumor cells are reactive with GFAP and vimentin, either diffusely or focally [6, 48, 54]. There is a second atypical cellular component consisting of smaller cells with prominent, more darkly staining nuclei and scant, sometimes barely visible cytoplasm. This is the so-called growth (proliferative) fraction which is MIB-1/Ki67 reactive [13, 39]. The larger eosinophilic cells show a much lower proliferation rate than the smaller component. Perivascular lymphocyte cuffing is variably present to some degree.

All three GA patients in this review had large hemispheric tumors detected by prenatal ultrasound or on the day of birth. The first patient in Table 8, a female of 32-week gestation, died at 2 months of age without treatment [6]. The GA occupied half of the intracranial cavity. Her histology showed no necrosis, vascular proliferation, or other cellular atypical features [6]. The second patient received surgery and chemotherapy and survived [54]. The third infant lived for 6 days without treatment. Her tumor cells exhibited mild pleomorphism, increased cellularity, a low MIB-1 index (<1 %), but palisading necrosis and endothelial proliferation were noted; this tumor was designated as malignant [48].

#### Treatment and outcome

The only survivor, patient 2 (tumor WHO grade 2), was a full-term male with a right occipital lobe primary [54] (Table 8). Microscopically, the GA revealed atypical tumor cells and an elevated MIB-1 index of 15 %. The child was alive and well even after a relapse at age 2.5 years. He was treated by complete surgical resection and chemotherapy consisting of vincristine and cis-platinum [54]. Current therapy of GA is discussed elsewhere [22, 26, 28, 65].

GA shows a tendency to behave aggressively more than other grade I and II astrocytomas. Some may progress to anaplastic astrocytoma and eventually GBM [54].

## Discussion

Astrocytoma is the foremost neuroglial tumor occurring throughout infancy and childhood and is derived from and composed of astrocytes showing varying degrees of differentiation. As a group, astrocytomas differ in their gross and histological features as well as in their site of origin and clinical manifestations [12, 15, 27, 30, 31, 37, 57, 68, 93] (Table 9 **)**.

Most perinatal astrocytomas occur outside the posterior cranial fossa and above the tentorium (Tables 2). When it can be determined, the cerebral hemisphere is the most common primary site. Those arising from this location tend to be large, involve more than one lobe, and displace the ventricle(s). In some instances, the brain may be almost completely replaced by tumor.

Astrocytoma typically presents in the fetus and neonate as an intracranial mass, which is the initial observation on prenatal or postnatal sonograms; hydrocephalus macrocephaly and intracranial hemorrhage, in that order, are additional presenting findings (Tables 2, 3, 4, 5, 6, 7, and 8).

Tumors vary considerably in their microscopic appearance. Histologically, they range from benign (low-grade) to malignant (high-grade) tumors (Table 9). Here, there were 55 high-grade astrocytomas compared to 46 low-grade ones [52].

Treatment is primarily surgical, like other brain tumors in this age group [31]. Sometimes surgery is preceded by a shunt, and in selected cases followed by chemotherapy [30, 31, 37]. Usually, irradiation is contraindicated because of immaturity of the developing brain and adjacent structures. The extent and size of the tumor and the general condition of the neonate are limiting factors that can preclude surgery. Some tumors can be inoperable because of their large size or unstable condition of the infant [31].
